# The miR-193a-3p-regulated ING5 gene activates the DNA damage response pathway and inhibits multi-chemoresistance in bladder cancer

**DOI:** 10.18632/oncotarget.3555

**Published:** 2015-03-12

**Authors:** Yang Li, Hui Deng, Lei Lv, Cheng Zhang, Liting Qian, Jun Xiao, Weidong Zhao, Qi Liu, Daming Zhang, Yingwei Wang, Jun Yan, Hongyu Zhang, Yinghua He, Jingde Zhu

**Affiliations:** ^1^ Department of Biology, School of Life Science, Anhui Medical University, Hefei, Anhui, China; ^2^ Cancer Epigenetics Program, Anhui Cancer Hospital, Hefei, Anhui, China; ^3^ Department of Urology, The First Affiliated Hospital of Harbin Medical University, Harbin, Heilongjiang Province, China; ^4^ Department of Radiotherapy, Anhui Cancer Hospital, Hefei, Anhui, China; ^5^ Department of Urology, Anhui Provincial Hospital, Hefei, Anhui, China; ^6^ Department of Gynecologic Cancer, Anhui Cancer Hospital, Hefei, Anhui, China; ^7^ School of Life Science and Technology, State Key Laboratory of Urban Water Resource and Environment, Harbin Institute of Technology, Harbin, Heilongjiang, China; ^8^ Department of Neurosurgery, The First Affiliated Hospital of Harbin Medical University, Harbin, Heilongjiang Province, China; ^9^ Department of Pathology, The First Affiliated Hospital of Harbin Medical University, Harbin, Heilongjiang Province, China; ^10^ Cancer Epigenetics Program, Shanghai Cancer Institute, Renji Hospital, Shanghai Jiaotong University, Shanghai, China

**Keywords:** ING5, miR-193a-3p, chemoresistance, bladder cancer, protein acetylation

## Abstract

As the major barrier to curative cancer chemotherapy, chemoresistance presents a formidable challenge to both cancer researchers and clinicians. We have previously shown that the bladder cancer (BCa) cell line 5637 is significantly more sensitive to the cytoxicity of five chemotherapeutic agents than H-bc cells. Using an RNA-seq-based omic analysis and validation at both the mRNA and protein levels, we found that the inhibitor of growth 5 (ING5) gene was upregulated in 5637 cells compared with H-bc cells, indicating that it has an inhibitory role in BCa chemoresistance. siRNA-mediated inhibition of ING5 increased the chemoresistance and inhibited the DNA damage response pathway in 5637 cells. Conversely, forced expression of EGFP-ING5 decreased the chemoresistance of and activated the DNA damage response pathway in H-bc cells. We also showed that ING5 gene expression is inhibited by miR-193a-3p and is instrumental in miR-193a-3p's role in activating BCa chemoresistance. Our results demonstrate both the role and mechanism of inhibition of BCa chemoresistance by ING5.

## INTRODUCTION

Bladder cancer (BCa) is the fourth-most common type of urogenital cancer in humans and the one that causes the greatest mortality [1]. It is notorious for both wide-spread resistance to drug treatments and high recurrence rates [2]. Although 50% of patients benefit from post-surgery chemotherapy [3], recurrent disease affects a high percentage of patients and is often resistant to 2^nd^-round chemotherapy [4], regardless of previous drug exposure [5]. Both intrinsic and acquired resistance to chemotherapeutics are common and vary drastically among cancer patients and among the different cancerous lesions or different regions of the same lesion within a single patient [6], making effective chemoresistance prediction and therapy impossible tasks.

Despite years of intensive effort, our understanding of multi-chemoresistance in cancer remains limited [7-10]. The mechanisms implicated include inefficient cellular drug uptake and accumulation, activation of the antioxidant glutathione system for detoxification, enhancement of DNA repair, and up-regulation of anti-apoptosis pathways [11-13]. The best-characterized genes that have been implicated in this multi-chemoresistance are ATP-binding cassette (ABC) transporters, such as P-glycoprotein (P-gp); breast cancer resistance proteins (BCRPs); and multi-drug resistance-associated proteins (MRPs) [14-16], which are over-expressed in multi-chemoresistant cancer cells [17]. Other protein-coding genes involved in BCa chemoresistance include ADAM10 [18], HIPK2 [19] and Nrf2 [20].

To find new protein-coding genes that are instrumental in BCa multi-chemoresistance, we conducted an RNA-seq-based omic analysis of a multi-chemosensitive BCa cell line (5637 cells) versus a resistant cell line (H-bc) [21] and identified 9051 differentially expressed protein-coding genes (not shown), including the inhibitor of growth 5 (ING5) gene. In the present study, we performed a systematic analysis of the ING5 gene for its role in BCa chemoresistance and its mechanisms of action. Furthermore, we showed that ING5 is a direct target of miR-193a-3p and mediates a significant part of miR-193a-3p's effect on multi-chemoresistance in BCa.

## RESULTS

### ING5 is a negative regulator of multi-chemoresistance in BCa cells

**Figure 1:**
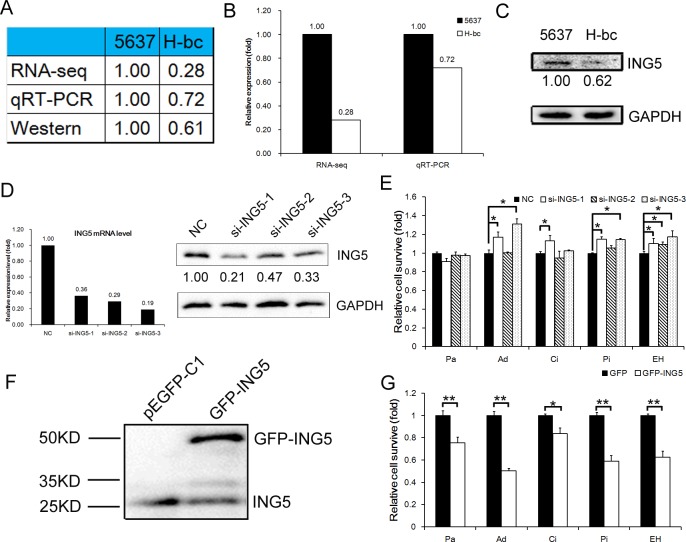
ING5 expression is negatively correlated with chemoresistance in BCa cells A, ING5 level in 5637 versus H-bc cells by RNA-seq, qRT-PCR, and Western blot analyses. B, Graph of RNA levels by RNA-seq and qRT-PCR. C, Western blot of ING5 in 5637 and H-bc cells. D, The ING5 mRNA (by qRT-PCR) and protein (by Western analysis) levels in the each of three ING5 siRNA (si-ING5-1, -2 and -3)-transfected versus the NC-transfected 5637 cells. E, The relative cell survival in the 5637 cells transfected with ING5 siRNAs vs. NC siRNA, assayed 72 hours after treatment with IC_50_-dosed drug (Pa, Ad, Ci, Pi or EH). F, The ING5 protein level in the EGFP-ING5-transfected versus the GFP (control)-transfected H-bc cells. G, The relative cell survival of the H-bc cells transfected with EGFP-ING5 versus GFP expression vector, assayed 72 hours after treatment with IC_50_-dosed drug. (*P<0.05; **P<0.01).

Among 9051 differentially expressed genes between a multi-chemosensitive (5637) and a resistant BCa cell line (H-bc) that were revealed by an RNA-seq-based omic study (data not shown), ING5 was among the top 10% most differentially expressed (Fig. 1A). The BCa chemoresistance-associated expression of the ING5 gene was confirmed by qRT-PCR analysis at the steady state mRNA level and a Western blot analysis at the protein level. The results showed that the ING5 protein (1.00:0.61, Fig. 1A and C) and mRNA expression levels were higher (RNA-seq-based omic: 1.00:0.28, and qRT-PCR analysis: 1.00:0.72, Fig. 1A and B) in 5637 cells than in H-bc cells.

To demonstrate its potential role in BCa chemoresistance, we suppressed ING5 mRNA expression to 33%, 47% and 21% of the NC control in 5637 cells by transfection of si-ING5-1, -2 and -3, respectively (Fig. 1D). The levels of drug-triggered cell death in the transfected cells at the IC_50_ of various drugs (paclitaxel, Pa; Adriamycin, Ad; cisplatin, Ci; pirarubicin, Pi; epirubicin hydrochloride, EH) was then determined. The most effective siRNA (si-ING5-1) relieved cell death in the 5637 cells stressed by all five drugs except Pa. The 2^nd^ most effective siRNA (si-ING5-3) failed to desensitize 5637 cells to the cell-killing effects of Pa and Ci, while the least effective siRNA (si-ING5-2) was only able to reduce cell death in the EH-treated 5637 cells. These observations suggest that ING5's effect on the drug type-specific chemoresistance of 5637 cells is sensitive to the expression of the ING5 gene within the cell (Fig. 1E). We further showed that forced upregulation of GFP-tagged ING5 protein (by Western blot analysis, Fig. 1F, and immunofluorescence analysis, Fig. S1) desensitized the transfected H-bc cells to the cell death triggered by all five drugs (Fig. 1G). In conclusion, ING5 had a significant effect on drug-triggered cell death for four (Ad, Ci, Pi and EH) of the five drugs tested in BCa cells.

### ING5 is a direct target of miR-193a-3p in BCa cells

The critical role of microRNA (miR) genes in the multi-chemoresistance of cancer has been well established [22]. The eminent examples for BCa chemoresistance are miR-30d, miR-181, miR-199a-5p [23] and miR-193a-3p [21, 24]. The DNA methylation-regulated miR-193a-3p confers 5-FU resistance to hepatocellular carcinoma (HCC) by repressing SRSF2 expression [24]. MiR-193a-3p also promotes multi-chemoresistance in BCa cells by suppressing SRSF2, HIC2 PLAU, LOXL4, PSEN1 and HOXC9 and altering the activities of five signaling pathways: DNA damage, Notch, NF-κB, Myc/Max and oxidative stress [21-24]. The miR-193a-3p level is significantly lower in 5637 than in H-bc cells, while its target genes SRSF2, HIC2 and PLAU are lower in H-bc than in 5637 cells [21], a pattern also displayed by ING5 in this pair of cell lines (Fig. 1A).

**Figure 2:**
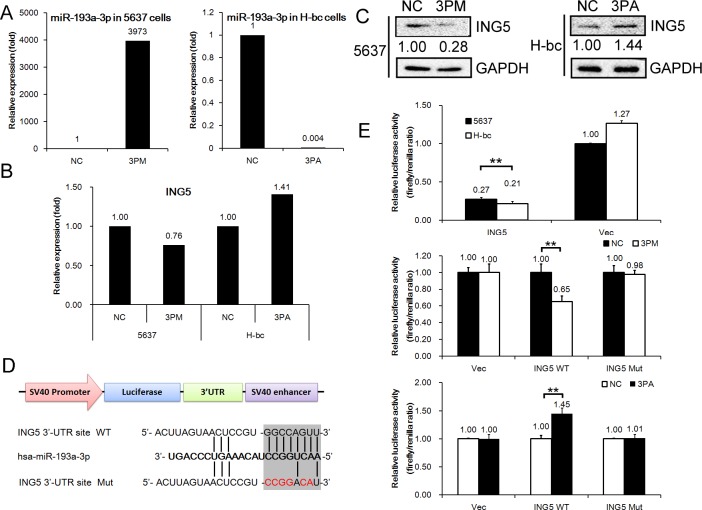
ING5 mRNA is a direct target of miR-193a-3p in BCa cells The levels of miR-193a-3p (A, by qRT-PCR) and ING5 mRNA (B, by qRT-PCR) and protein (C, by Western analysis) expression in the miR-193a-3p mimic (3PM)-transfected 5637 cells and the miR-193a-3p antagomiR (3PA)-transfected H-bc cells versus the negative control (NC)-transfected cells. D, The miR-193a-3p target sequences (the wild-type (WT) and mutant (Mut) in shadow) in the 3′-UTR of the ING5 gene. E, The relative luciferase activity (fold change) of the reporters with wild-type (WT) UTR, mutant (Mut) UTR or UTR minus (Vec) in the miR-193a-3p mimic (3PM)-transfected 5637 or antagomiR (3PA)-transfected H-bc or Mock (NC)-transfected cells. The Renilla luciferase activity of the co-transfected reporter construct was determined to control for the transfection efficiency. A representative result from three independent experiments is shown. Error bars represent the s.e.m. **P<0.05; **P<0.01 by Student's t-test.

Incidentally, ING5 was on the list of miR-193a-3p targets suggested by TargetScan software (http://www.targetscan.org/). Therefore, we determined the ING5 level in miR-193a-3p mimic-transfected 5637 and antagomiR-transfected H-bc versus NC (a scrambled-sequence control)-transfected cells. In parallel with changes in the miR-193a-3p level (Fig. 2A), miR-193a-3p mimic transfection suppressed ING5 mRNA expression to nearly 76% (Fig. 2B) and protein expression to 25% (Fig. 2C) of the NC level in 5637 cells. Transfection with the miR-193a-3p antagomiR raised the ING5 mRNA level to 141% (Fig. 2B) and the protein level to 145% of the level in the NC-transfected H-bc cells (Fig. 2C).

Next, we inserted the ING5 UTR (1-1037 bp) with either a wild-type or mutant miR-193-3p target sequence downstream of the firefly luciferase gene into the pGL3-control vector (Promega) to create the pGL3-ING5 UTR WT or the pGL3-ING5 UTR Mut construct, respectively (Fig. 2D). The pGL3-ING5 UTR WT, pGL3-ING5 UTR Mut and pGL3 constructs were individually transfected into 5637 and H-bc cells. The firefly luciferase activity was measured to determine whether the differentially expressed miR-193a-3p in this pair of cell lines was functional. As shown in Fig. 2E, pGL3-ING5-UTR WT but not the other 2 reporter constructs produced significantly higher luciferase activity in 5637 than in H-bc cells. Furthermore, only the luciferase activity of pGL3-ING5-UTR WT was suppressed in the mimic-transfected 5637 cells and upregulated in the antagomiR-transfected H-bc cells relative to the pGL3 control (Fig. 2E). In conclusion, the ING5 gene is a direct post-transcriptional target of miR-193-3p.

**Figure 3:**
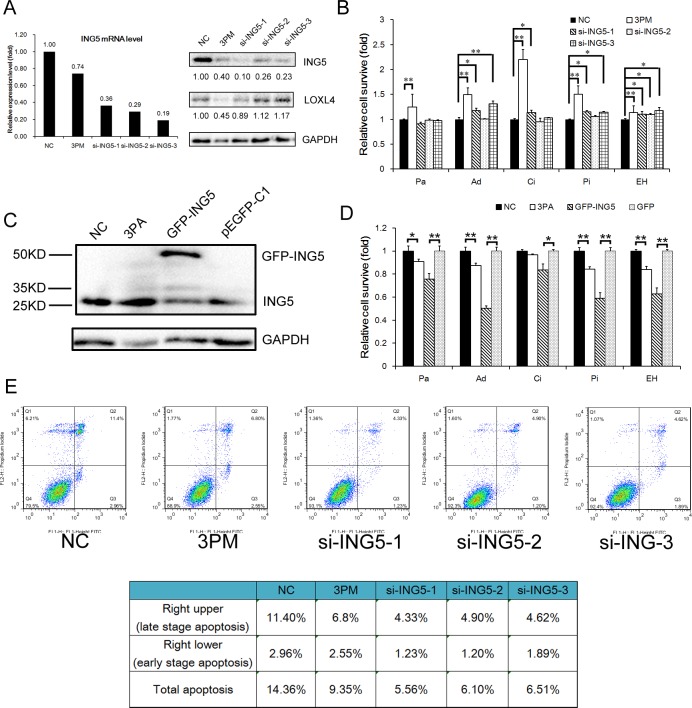
ING5's effects on drug-triggered cell death and apoptosis in BCa cells A, The ING5 and LOXL4 protein levels (Western analysis) in the 3PM or ING5 siRNA (si-ING5-1, 2 and 3)-transfected versus the NC-transfected 5637 cells. B, The changes in cell survival of the 5637 cells transfected by miR-193a-3p mimic (3PM) or each ING5 siRNA over the NC-transfected 5637 cells, 72 hours after treatment with IC_50_-dosed drug (Pa, Ad, Ci, Pi or EH). The ING5 protein level (Western blot analysis C, and the effect on the levels of drug-triggered cell death D, in the 3PA- or EGFP-ING5 construct-transfected versus the NC- or EGFP-transfected H-bc cells. E, Reduced apoptotic effects by miR-193a-3p mimic- or si-ING5-transfected 5637 cells revealed by FACS analysis: the percentage of apoptotic cells (late and early stage) is also summarized in the table. *P<0.05; **P<0.01.

We further compared the levels of drug-triggered cell death in 5637 cells transfected with miR-193a-3p mimic versus ING5 siRNAs. Whereas miR-193a-3p reduced the ING5 level to 40%, all three si-RNAs reduced the ING5 protein level to no more than 30% of the NC control and had no suppressive effects on the LOXL4 protein, another direct target of miR-193a-3p (Fig. 3A). The ING5 mRNA level was also reduced by miR-193a-3p and the siRNAs to comparable degrees (Fig. 3A). Although the miR-193a-3p mimic had a relatively weaker repressive effect on ING5, the mimic-transfected 5637 cells suffered less drug-triggered death in response to all five IC_50_-dosed drugs (Fig. 3B), while only the most potent siRNA (si-ING5-1) significantly (but to a reduced extent in the cells stressed by Ad, Ci or Pi) decreased the level of cell death in 5637 cells stressed by Ad, Ci, Pi or EH, but not Pa (Fig. 3B). The observed disparity between the miR-193a-3p mimic and the most effective siRNA against ING5 (si-ING5-1) in terms of drug-triggered cell death is likely attributable to the downregulation of other miR-193a-3p target proteins, such as SRSF2, PLAU, HIC2 and LOXL4 (shown in Fig. 3A) in mimic-transfected but not in si-ING5-1-transfected 5637 cells (Fig. 3B). Incidentally, the siRNA (−1, −2 and −3, respectively)-mediated ING5 repression reduced the percentage of late-apoptotic (and necrotic) cells from 11.4% (the right upper quadrant in Fig. 3C) to 4.33%, 4.90%, and 4.62% and decreased the percentage of early-stage apoptotic cells (the right lower quadrant) from 2.96% to 1.23%, 1.20% and 1.89% (Fig. 3C and 3E). Intriguingly, although miR-193a-3p mimic had the strongest resistance-promoting effect (Fig. 3B), it had a lower anti-apoptotic effect than the ING5 siRNAs (Fig. 3C), implying that other mechanisms, including G2 arrest, may be involved. Indeed, we have previously shown that miR-193a-3p mimic causes G2 arrest in 5637 cells [25, 26], an effect that was not observed in ING5 siRNA-transfected 5637 cells (Supplementary Fig. S2). In the reverse experiment, the miR-193a-3p antagomiR sensitized the H-bc cells to the cytotoxicity caused by all five drugs, as did the GFP-ING5 expression constructs, to an even greater extent (Fig. 3C and D).

All of these observations suggest that ING5 acts as the downstream target of miR-193a-3p, mediating a significant part of miR-193a-3p's effect on BCa chemoresistance to Ad, Ci, Pi and EH in 5637 cells (Fig. 1E and 3B) or to all five drugs in H-bc cells (Figs. 1G and 3D).

### ING5 positively regulates the DNA damage response pathway in the context of BCa multi-chemoresistance

**Figure 4:**
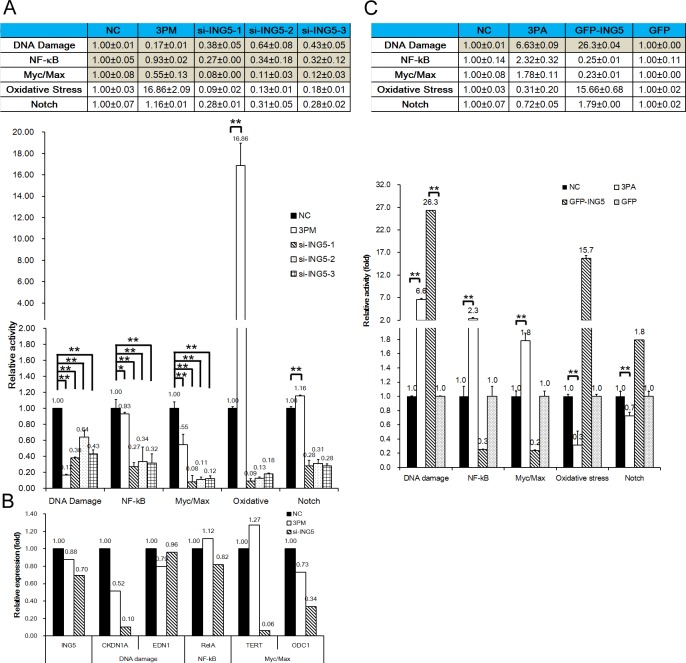
ING5 ‘s effect on five signaling pathways in BCa cells, assayed using a Qiagen pathway reporter system A, The relative activities (mean ± S.D) of the DNA damage, NF-κB, Myc/Max, oxidative stress and Notch pathways in the ING5 siRNA- or miR-193a-3p mimic-transfected versus the NC-transfected 5637 cells. B, The mRNA levels (qRT-PCR) of ING5, a downstream target gene of the DNA damage, NF-κB and Myc/Max pathways and a component of the gamma-secretase complex in the miR-193a-3p mimic- or ING5 siRNA-transfected 5637 cells. C, The relative activities (mean ± S.D) of five pathways in the EGFP-ING5- or miR-193s-3p antagomiR (3PA)-transfected versus the NC-transfected H-bc cells. *P<0.05.

We recently reported that miR-193a-3p regulates multi-chemoresistance by repressing the expression of three of its downstream targets (SRSF2, PLAU, HIC2, LOXL4, PSEN1 and HOXC9) and in turn altering the activities of five signaling pathways: DNA damage response, Notch, NF-κB, Myc/Max and oxidative stress [21-24]. It is desirable to determine which of these five pathways are also affected by the forced changes in the ING5 level in both 5637 and H-bc cells. In parallel with the reduced ING5 levels (Fig. 3A), the activities of the DNA damage response, NF-κB and Myc/Max pathways were repressed by the ING5 siRNAs in a similar fashion to miR-193a-3p mimic, but they differed in potency. For example, the activity of the DNA damage response pathway was reduced by ING5 siRNAs-1, -2 and -3 to 43%, 64% and 48% of the NC level, respectively, but miR-193a-3p mimic reduced the activity to 17% of the NC level. We further assessed the changes in the mRNA levels of several genes in 5637 cells transfected with siRNA compared with the NC- and miR-193a-3p mimic-transfected cells, including the downstream gene targets of the DNA damage response pathway (CDKN1A and EDN1) NF-κB (RelA) pathway, and Myc/Max pathway (TERT and ODC1) as well as ING5. In parallel with the reduced ING5 mRNA expression levels, both the CDKN1A and ODC1 mRNA levels were downregulated (Fig. 4B). Therefore, the ING5 connection to miR-193a-3p in 5637 cells in the context of BCa multi-chemoresistance (Figs. 1, 2, 3) is most likely attributable to its effect on the activities of these three pathways that are regulated by miR-193a-3p.

Last in this line of experiments, we transfected H-bc cells with a GFP-ING5 expression construct or a GFP (negative control) construct to overexpress ING5 (Fig. 3C). Only the activity of the DNA damage response pathway among the pathways examined in 5637 cells (Fig. 4A) was activated dramatically by both miR-193a-3p antagomiR and EGP-ING5 overexpression (Fig. 4C). Therefore, ING5's role in mediating miR-193a-3p's effect on BCa chemoresistance is principally accomplished by its impact on the DNA damage response pathway.

### ING5 expression is reduced in miR-193a-3p agomiR-injected 5637 tumor xenografts and increased in antagomiR-injected H-bc tumor xenografts in nude mice

**Figure 5:**
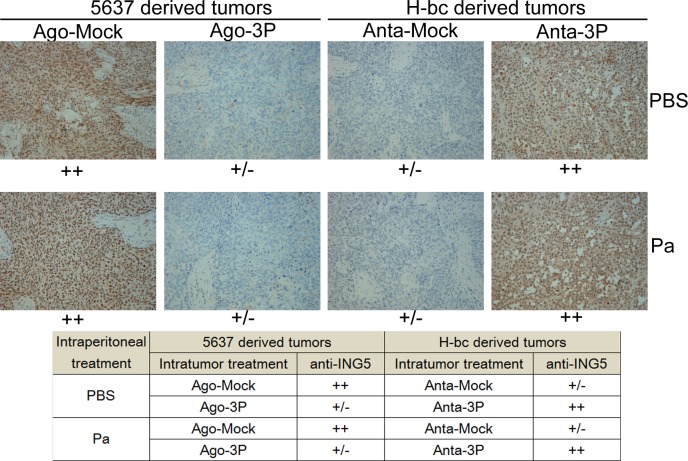
The ING5 level (immunohistochemical staining) in tissue slides of the miR-193a-3p agomiR-injected 5637 and miR-193a-3p antagomiR-injected H-bc tumor xenografts versus the NC-injected tumor xenografts The levels of ING5 protein in each group are summarized in the table.

In our previous study, we showed that miR-193a-3p promotes Pa chemoresistance in BCa cells in tumor xenografts of nude mice by repressing three of its targets in the tumor tissues: SRSF2, PLAU and HIC2 [21]. In the present study, we semi-quantified the ING5 levels (Fig. 5) in the same set of the xenografts by immunohistological analysis. The injection of either miR-193a-3p agomiR into 5637 tumor xenografts or of the antagomiR into H-bc tumor xenografts indeed led the opposite changes of ING5 protein overexpression in the tumor tissues (Fig. 5). This observation further strengthens the notion that ING5 has a role in miR-193a-3p's effect promoting Pa-chemoresistance in BCa cells.

## DISCUSSION

Despite years of intensive research efforts, the underlying mechanisms of cancer multi-chemoresistance remain elusive. To identify new protein-coding genes that are instrumental to BCa multi-chemoresistance, we performed a comparative RNA-seq omic analysis of a multi-chemosensitive (5637) and a resistant cell line (H-bc) and identified a panel of differentially expressed genes. The ING5 gene, which was more highly expressed in 5637 than in H-bc cells, was systematically studied in both cultured cells (Figs. 1, 2, 3, 4) and tumor xenografts in nude mice (Fig. 5). The ING5 gene is a direct target of miR-193a-3p (Figs. 2, 3, 4, 5). In forced-expression experiments, ING5 improved cell survival in response to four (in 5637 cells) or five drugs (in H-bc cells), essentially mimicking the effect of miR-193a-3p (Fig. 3). In agreement with a previous report showing that forced knockdown of ING5 reduces cell apoptosis [27], the percentage of apoptotic cells was reduced in the ING5 siRNA-transfected 5637 cells to a greater extent than following transfection with a miR-193a-3p mimic (Fig. 3E). However, the G2 arrest effect of miR-193a-3p was not observed in si-ING5-s1-transfected 5637 cells (Fig. S2). Furthermore, only the DNA damage response pathway among the five pathways affected by miR-193a-3p was influenced by the changed level of ING5 protein in both 5637 and H-bc cells (Fig. 4). MiR-193a-3p potently promotes multi-chemoresistance in both HCC [24] and BCa by repressing its three downstream targets: SRSF2, PLAU, HIC2, LOXL4, PSEN1 and HOXC9 [21-24]. siRNA-mediated repression of each of these three genes only partially reproduces the effects of miR-193a-3p mimic on 5637 cells in terms of drug-triggered cell death and pathway activity [21]. The data suggest that the collective effects of the reduced levels of those three proteins as well as ING5 (in this report) contribute to miR-193a-3p's impact on BCa chemoresistance, which cannot be fully reproduced by altering the level of any single target in 5637 or H-bc cells. It is therefore not unexpected that the most effective siRNA against ING5 repressed ING5 protein more than miR-193a-3p did but relieved drug-triggered cell death to a lesser extent in the transfected 5637 cells in both a drug type-specific and quantitative manner (Fig. 3B).

ING5 is the most recently discovered and least characterized member of the inhibitor of growth (ING) family. It is mutated at a significant frequency in all types of cancers examined (COSMIC http://cancer.sanger.ac.uk]). A network analysis performed by combining literature data with an analysis of the public protein-protein interaction databases Wiki-PI [http://severus.dbmi.pitt.edu/wiki-pi/] and BioGRID 3.2 [http://thebiogrid.org/] has linked the ING5 gene to the DNA damage pathway. ING5 directly interacts with p53 and EP300 (http://severus.dbmi.pitt.edu/wiki-pi/index.php/search?q=ING5 and http://thebiogrid.org/124016/summary/homo-sapiens/ing5.html) and acts as a key component of the HBO1-containing HAT complex that increases the acetylation states of both histone and nonhistone proteins such as p53 [28, 29]. It promotes p53-dependent pathways and in turn induces apoptosis and negatively regulates tumorigenesis [27, 30-32]. In this context, ING5 acts as an important cofactor of the Tip60 (the catalytic subunit of the NuA4 histone acetyltransferase complex)-centered complex that increases the K120 acetylation of p53 in stressed cells where apoptosis and/or cell-cycle arrest are induced [27, 33]. ING5 can bind with p53 and EP300, a component of the histone acetyl transferase complex, suggesting that it is involved in a p53-dependent regulatory pathway [33]. Indeed, the DNA damage response pathway with p53 as the master transcription factor is the principal pathway affected by both ING5 and its upstream regulator miR-193a-3p (Fig. 4). The other two pathways likely to be regulated are NF-κB and Myc/Max (Fig. 4A), as suggested by our observations in 5637 cells but not in H-bc cells (Fig. 4C). By qRT-PCR analysis, we showed that CDKN1A (a target of the DNA damage response pathway), RelA (a target of the NF-κB pathway), and TERT and ODC1 (targets of the Myc/Max pathway) were downregulated in ING5 siRNA-transfected 5637 cells (Fig. 4C). Moreover, TIP60 is involved in transcriptional activation principally by acetylating nucleosomal histones, and it interacts with the NF-κB RelA/p65 subunit to increase the transcriptional activity of p65 through protein-protein interaction [34]. The NF-κB pathway is functionally antagonistic to p53 in multiple tumor types [35, 36]. Moreover, the transcriptional activation by c-MYC relies in part on its ability to recruit the TIP60-containing complexes and the subsequent acetylation [37]. As a crucial inhibitor of p53, MDM2 is transcriptionally activated by both NF-κB and p53 and binds/inhibits the p65 RelA subunit of NF-κB [38]. Additionally, there are reports of functional links between the ING5 gene and the Myc/Max pathway, chiefly involving p53 as a critical mediator within the ARF–Mdm2–p53 regulatory loop [39-41].

ING5 contains a PHD-finger domain, a common motif in proteins with a chromatin remodeling function [42], aiding in the acetylation of the core histones [43]. However, neither miR-193a-3p mimic transfection nor the knockdown of ING5 in 5637 cells reduced the overall acetylation level of histone H3 (Supplementary Fig. S3). More research is needed to better understand ING5's role in the control of the overall acetylation state of the core histones and therefore genomic transcriptional activities.

**Figure 6:**
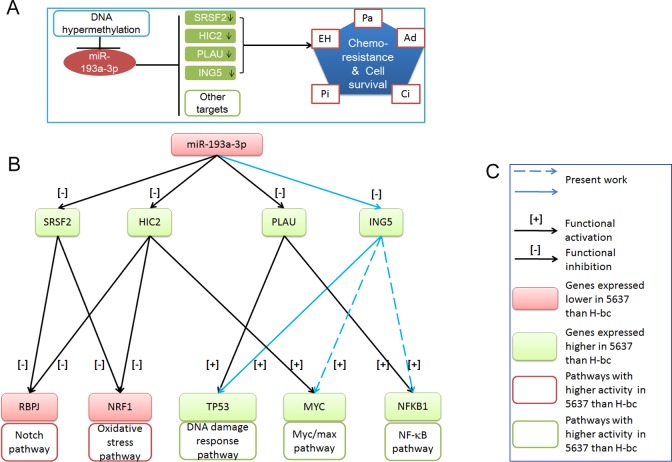
The working model for miR-193a-3p's role in the regulation of chemoresistance in BCa A, SRSF2, HIC2, PLAU (previous work) and ING5 (present work) are under the negative regulation of the DNA methylation-regulated miR-193a-3p. Their downregulation is correlated with chemoresistance to four of the indicated chemotherapeutics. B, miR-193a-3p's effect on the signaling pathways *via* its repression of SRSF2, HIC2 and PLAU (previous work) and ING5 (this work). C, Figure legend.

In summary, we showed that the ING5 gene, together with other downstream genes (SRSF2, PLAU and HIC2), mediates a significant part of miR-193a-3p's positive impact on multi-chemoresistance in BCa cells, mainly via its positive impact on the DNA damage response pathway (summarized in Fig. 6).

## METHODS

### Cell lines

BCa cell lines were purchased from the Chinese Academy of Cell Resource Center (Shanghai, China): 5637 (ATCC NO. HTB-9) and H-bc (established by the Cancer Research Institute of Kunming Medical College, 1986). Both cell lines were cultured in RPMI 1640 (Invitrogen, USA) plus 10% fetal bovine serum (Invitrogen, USA) and 1% glutamine at 37°C in 5% CO_2_.

### Reagents for the transient transfection assays

The mimic, agomiRs, antagomiRs, siRNAs, the scrambled sequence (negative control, NC) and the riboFECT CP transfection kit were supplied by Ribobio (Guangzhou, China). The GFP-tagged overexpression ING5 construct (construct from pReciever-M98) was purchased from Genecopia, (Guangzhou, China). Transfection of both the ribonucleic acid reagents mentioned above and the reporter plasmids was performed according to the manufacturer's instructions.

Chemically modified mimic oligonucleotides (agomiRs) were synthesized to regulate miR-193a-3p/5p expression *in vivo*. The 3′ end of the oligonucleotides was conjugated to cholesterol, and all of the bases were 2′-OMe modified. The agomiR oligonucleotides were deprotected, desalted and purified by high-performance liquid chromatography.

The siRNA sequences used for ING5 interference in this study were as follows:

si-ING5-1:

5′ CCAUGUACUUGGAGCACUAdTdT 3′

3′ dTdTGGUACAUGAACCUCGUGAU 5′

si-ING5-2:

5′ GGAAUACAGUGACGACAAAdTdT 3′

3′ dTdTCCUUAUGUCACUGCUGUUU 5′

si-ING5-3:

5′ CCUACGAGAUGGUGGAUAAdTdT 3′

3′ dTdTGGAUGCUCUACCACCUAUU 5′

### Luciferase reporter assay

A full-length human ING5 3′-UTR (1037 bp) with a wild-type or a mutant target sequence for miR-193a-3p was cloned into the 3′ flank of the luciferase coding sequence of pGL3 (Invitrogen) to construct pGL3-luc-ING5 WT or pGL3-luc-ING5 Mut, respectively. All of the constructs were confirmed by DNA sequencing. Cells were seeded into 96-well plates at approximately 1×10^4^ cells per well and transfected with a mixture of 50 ng of pGL3-luc-ING5 WT or Mut, 5 ng of Renilla plus 5 pmol of mimic or NC nucleotide using the riboFECT CP transfection kit according to the manufacturer's instructions. Both the firefly and Renilla luciferase activities were measured 18 hours after transfection by the Dual-Luciferase Reporter Assay System (Promega) using a Promega GloMax 20/20 luminometer. The relative firefly luciferase activities of the UTR constructs and pathway reporter constructs were analyzed as previously reported [21].

### Chemoresistance profiling (IC_50_ determination)

Clinical-grade (NCI Dictionary of Cancer Terms, http://www.cancer.gov/dictionary), pirarubicin (Pi, Wanle, Shenzhen) paclitaxel (Pa, Taiji, Sichuan), Adriamycin (Ad, Pfizer, Jiangsu), epirubicin hydrochloride (EH, Haizheng, Zhejiang), and cisplatin (Ci, Haosen, Jiangsu) were used. Relative IC_50_ values were determined as previously described [21].

### Apoptosis analysis

Cells were harvested and rinsed with phosphate-buffered saline (PBS) twice. Then, 5 μl of fluorescein isothiocyanate (FITC)-labeled enhanced Annexin V and 5 μl (20 μg/ml) of propidium iodide were added to the 100 μl cell suspension. Following incubation in the dark for 15 min at room temperature, the samples were diluted with 400 μl PBS. Flow cytometry was carried out on a FACSCalibur instrument. The results were analyzed according to the manufacturer's instructions. The experiments were performed independently three times, and a representative result is shown herein.

### RNA analysis

Total RNA was isolated from the cells at the logarithmic phase using TRIzol reagent (Tiangen Biotech Co., Ltd., Beijing, China). For mRNA analysis, cDNA primed by oligo-dT was generated using a PrimeScript RT reagent kit (Tiangen Biotech Co., Ltd., Beijing, China), and the mRNA levels of the genes were quantified by duplex-qRT-PCR analysis using TaqMan probes with a different fluorescence for the β-actin (provided by Shing Gene, Shanghai, China) and a FTC-3000P PCR instrument (FUNGLYN BIOTECH INC, Canada). Using the 2^−ΔΔCt^ method, gene expression was normalized to β-actin and then compared between groups. The sequences of the primers and probes used for the qRT-PCR analysis were as follows:

hING5 F: 5′-TCCAGAACGCCTACAGCAAG-3′
hING5 R: 5′-TGCCCTCCATCTTGTCCTTC-3′
hING5 probe: 5′-CY5-CGACAAAGTGCAGCTG GCCATGC-3′
hACTB F: 5′-GCCCATCTACGAGGGGTATG-3′
hACTB R: 5′-GAGGTAGTCAGTCAGGTCCCG-3′
hACTB probe: 5′HEX-CCCCCATGCCATCCTG CGTC-3′
hCDKN1A F: 5′-CACTGTCTTGTACCCTTGT GCC-3′
hCDKN1A R: 5′-GGCTTCCTCTTGGAGAAGA TCA-3′
hCDKN1A probe: 5′-ROX-CCCCAGGTGGACCT GGAGACTCTC-3′
hEDN1 F: 5′-CTTCTGCCACCTGGACATCA-3′
hEDN1 R: 5′-CATCTATTTTCACGGTCTG TTGC-3′
hEDN1 probe: 5′-ROX-CGTTGTTCCGTATGG ACTTGGAAGCC-3′
hRelA F: 5′-ATGGCTTCTATGAGGCTGAGC-3′
hRelA R: 5′-AGGGGTTGTTGTTGGTCTGG-3′
hRelA probe: 5′-ROX-CGGACCGCTGCATCCA CAGTTTC-3′
hTERT F: 5′-GCTGCTCAGGTCTTTCTTTT ATG-3′
hTERT R: 5′-ACCTCTGCTTCCGACAGCTC-3′
hTERT probe: 5′-ROX-CGGAAGAGTGTCTG GAGCAAGTTGC-3′
hODC1 F: 5′-ATGATAGCAAAGCCATCGTGA-3′
hODC1 R: 5′-CCCAGACTCTGCACCAACTG-3′
hODC1 probe: 5′-ROX-CTACCGGGACAG GATTTGACTGTGC-3′

### Bulge-Loop™ miRNA qRT-PCR

For detecting and quantifying the expression of specific miRNAs, RNA was reverse-transcribed using the Bulge-Loop™ miRNA qRT-PCR Primer Set (Ribobio) and quantified by SYBR Green-based real-time PCR analysis in a FTC-3000P (FUNGLYN BIOTECH INC, Canada). The Ct values of the target miRNAs were normalized to the Ct values of U6 RNA before quantification using the 2-^ΔΔCt^ method.

### Western blot

Cells were lysed with a lysis buffer (60 mM Tris-HCl, pH 6.8, 2% sodium dodecyl sulfate [SDS], 20% glycerol, 0.25% bromophenol blue, 1.25% 2-mercaptoethanol) and heated at 100°C for 10 min before electrophoresis/Western blot analysis. The anti-ING5 antibody (10665-1-AP) was purchased from Proteintech, and anti-GAPDH antibody (AM1020a), anti-rabbit IgG peroxidase-conjugated antibody (LP1001b), and HRP-conjugated goat anti-mouse IgG antibody (LP1002a) were provided by Wuxiphama, Shanghai, China. The target bands were revealed by an enhanced chemiluminescence reaction (Pierce), and the relative density of each protein over GAPDH was quantified using a Gel-Pro Analyzer (Media Cybernetics).

### *In vivo* studies

Animal experiments were performed as previously described [21]. ING5 protein expression was measured using immunohistochemical analysis on 5-mm slices of formalin-fixed paraffin-embedded tumor xenografts in nude mice. To avoid inter-treatment bias, the tissue slides from all six groups were made on a single slide and subjected to the same immunostaining simultaneously. Antigens were retrieved by pretreating dewaxed sections in a microwave oven at 750 W for 5 min in a citrate buffer (pH 6) processed with the Super Sensitive Link-Labeled Detection System (Biogenex, Menarini, Florence, Italy). The enzymatic activities were developed using 3-amino-9-ethylcarbazole (Dako, Milan, Italy) as a chromogenic substrate. Following counterstaining with Mayer's hematoxylin (Invitrogen), slides were mounted in aqueous mounting medium (glycergel, Dako). Pictures were taken using a LEICA DM 4000B microscope, while the relative level of each protein was calculated using LEICA software. The percentage of the mock- over the chemotherapeutic-treated tumors was calculated and plotted.

### Statistical analysis

Data are presented as the means, and error bars indicate the standard deviation (S.D.) or standard error (S.E.). All of the statistical analyses were performed using Excel (Microsoft, Redmond, WA) or Prism (GraphPad Software Inc., La Jolla, CA). The two-tailed Student's t-test, one-way analysis of variance or the Mann-Whitney U test was used to calculate statistical significance. A P-value of <0.05 was considered significant.

## SUPPLEMENTARY MATERIALS AND FIGURES



## Abbreviations

BCa: bladder cancer
MiR: microRNA
HCC: hepatocellular cancer
Pi: pirarubicin
Pa: paclitaxel
Ad: Adriamycin
EH: epirubicin hydrochloride
Ci: cisplatin
5-FU: 5-fluorouracil
UTR: untranslated region
ING5: inhibitor of growth family, member 5
TERT: telomerase reverse transcriptase
ODC1: ornithine decarboxylase 1
CDKN1A: cyclin-dependent kinase inhibitor 1A (p21, Cip1)
EDN1: endothelin 1
RelA: v-rel avian reticuloendotheliosis viral oncogene homolog A, p65

## References

[1] Siegel R, Ma J, Zou Z, Jemal A (2014). Cancer statistics, 2014. CA: a cancer journal for clinicians.

[2] von der Maase H, Sengelov L, Roberts JT, Ricci S, Dogliotti L, Oliver T, Moore MJ, Zimmermann A, Arning M (2005). Long-term survival results of a randomized trial comparing gemcitabine plus cisplatin, with methotrexate, vinblastine, doxorubicin, plus cisplatin in patients with bladder cancer. Journal of Clinical Oncology.

[3] Cimino GD, Pan C-x, Henderson PT (2013). Personalized medicine for targeted and platinum-based chemotherapy of lung and bladder cancer. Bioanalysis.

[4] Chang JS, Lara PN, Pan C-X (2012). Progress in personalizing chemotherapy for bladder cancer. Advances in urology.

[5] Gordon RR, Nelson PS (2012). Cellular senescence and cancer chemotherapy resistance. Drug Resistance Updates.

[6] Gerlinger M, Rowan AJ, Horswell S, Larkin J, Endesfelder D, Gronroos E, Martinez P, Matthews N, Stewart A, Tarpey P (2012). Intratumor heterogeneity and branched evolution revealed by multiregion sequencing. New England Journal of Medicine.

[7] JG Marin J, Briz O, J Monte M, G Blazquez A, IR Macias R (2012). Genetic variants in genes involved in mechanisms of chemoresistance to anticancer drugs. Current cancer drug targets.

[8] Li F, Sethi G (2010). Targeting transcription factor NF-κB to overcome chemoresistance and radioresistance in cancer therapy. Biochimica Et Biophysica Acta (BBA)-Reviews on Cancer.

[9] Capaccione KM, Pine SR (2013). The Notch signaling pathway as a mediator of tumor survival. Carcinogenesis.

[10] Abbotts R, Thompson N, Madhusudan S (2014). DNA repair in cancer: emerging targets for personalized therapy. Cancer management and research.

[11] Landriscina M, Maddalena F, Laudiero G, Esposito F (2009). Adaptation to oxidative stress, chemoresistance, and cell survival. Antioxidants & redox signaling.

[12] Ahmad S (2010). Platinum–DNA interactions and subsequent cellular processes controlling sensitivity to anticancer platinum complexes. Chemistry & biodiversity.

[13] Sau A, Pellizzari Tregno F, Valentino F, Federici G, Caccuri AM (2010). Glutathione transferases and development of new principles to overcome drug resistance. Archives of biochemistry and biophysics.

[14] Leslie EM, Deeley RG, Cole SP (2005). Multidrug resistance proteins: role of P-glycoprotein, MRP1, MRP2, and BCRP (ABCG2) in tissue defense. Toxicology and applied pharmacology.

[15] Borst P, Evers R, Kool M, Wijnholds J (2000). A family of drug transporters: the multidrug resistance-associated proteins. Journal of the National Cancer Institute.

[16] Tan B, Piwnica-Worms D, Ratner L (2000). Multidrug resistance transporters and modulation. Current opinion in oncology.

[17] Jedlitschky G, Leier I, Buchholz U, Barnouin K, Kurz G, Keppler D (1996). Transport of glutathione, glucuronate, and sulfate conjugates by the MRP gene-encoded conjugate export pump. Cancer research.

[18] Fu L, Liu N, Han Y, Xie C, Li Q, Wang E (2014). ADAM10 regulates proliferation, invasion, and chemoresistance of bladder cancer cells. Tumor Biology.

[19] Lin J, Zhang Q, Lu Y, Xue W, Xu Y, Zhu Y, Hu X (2014). Downregulation of HIPK2 Increases Resistance of Bladder Cancer Cell to Cisplatin by Regulating Wip1. PloS one.

[20] Hayden A, Douglas J, Sommerlad M, Andrews L, Gould K, Hussain S, Thomas GJ, Packham G and, Crabb SJ (2014). The Nrf2 transcription factor contributes to resistance to cisplatin in bladder cancer. Urologic Oncology: Seminars and Original Investigations.

[21] L Lv HD, Y Li, C Zhang, X Liu, Q Liu, D Zhang, L Wang, Y Pu, H Zhang, Y He, Y Wang, Y Yu, T Yu, J Zhu (2014). The DNA methylation-regulated miR-193a-3p dictates the multi-chemoresistance of bladder cancer via repression of SRSF2/PLAU/HIC2 expression. Cell Death & Disease.

[22] Lei Lv, Yang Li, Hui Deng, Cheng Zhang, Youguang Pu, Liting Qian, Jun Xiao, Weidong Zhao, Qi Liu, Daming Zhang, Yingwei Wang, Hongyu Zhang, Yinghua He, Jingde Zhu (2015 Feb 1). ” MiR-193a-3p promotes the multi-chemoresistance of bladder cancer by targeting the HOXC9 gene.”. Cancer Lett.

[23] Hui Deng, Lei Lv, Yang Li, Cheng Zhang, Fang Meng, Youguang Pu, Jun Xiao, Liting Qian, Weidong Zhao, Qi Liu, Daming Zhang, Yingwei Wang, Hongyu Zhang, Yinghua He, Jingde Zhu (2014 Dec 24). “The miR-193a-3p regulated PSEN1 gene suppresses the multi-chemoresistance of bladder cancer.”. Biochim Biophys Acta.

[24] Hui Deng, Lei Lv, Yang Li, Cheng Zhang, Fang Meng, Youguang Pu, Jun Xiao, Liting Qian, Weidong Zhao, Qi Liu, Daming Zhang, Yingwei Wang, Hongyu Zhang, Yinghua He, Jingde Zhu (2014 Oct 14). “miR-193a-3p regulates the multi-drug resistance of bladder cancer by targeting the LOXL4 gene and the Oxidative Stress pathway.”. Molecular Cancer.

[25] Deng H, Lv L, Li Y, Zhang C, Meng F, Pu Y, Xiao J, Qian L, Zhao W, Liu Q, Zhang D, Wang Y, Zhang H, He Y, Zhu J (2014). The miR-193a-3p regulated PSEN1 gene suppresses the multi-chemoresistance of bladder cancer. Biochimica et biophysica acta.

[26] Lv L, Li Y, Deng H, Zhang C, Pu Y, Qian L, Xiao J, Zhao W, Liu Q, Zhang D, Wang Y, Zhang H, He Y, Zhu J (2015). MiR-193a-3p promotes the multi-chemoresistance of bladder cancer by targeting the HOXC9 gene. Cancer Lett.

[27] Liu N, Wang J, Wang J, Wang R, Liu Z, Yu Y, Lu H (2013). ING5 Is a Tip60 cofactor that acetylates p53 in response to DNA damage. Cancer research.

[28] Doyon Y, Cayrou C, Ullah M, Landry A-J, Côté V, Selleck W, Lane WS, Tan S, Yang X-J, Côté J (2006). ING tumor suppressor proteins are critical regulators of chromatin acetylation required for genome expression and perpetuation. Molecular cell.

[29] Iizuka M, Sarmento OF, Sekiya T, Scrable H, Allis CD, Smith MM (2008). Hbo1 Links p53-dependent stress signaling to DNA replication licensing. Molecular and cellular biology.

[30] Coles AH, Jones SN (2009). The ING gene family in the regulation of cell growth and tumorigenesis. Journal of cellular physiology.

[31] Jafarnejad SM, Li G (2012). Regulation of p53 by ING family members in suppression of tumor initiation and progression. Cancer and Metastasis Reviews.

[32] Guérillon C, Larrieu D, Pedeux R (2013). ING1 and ING2: multifaceted tumor suppressor genes. Cellular and Molecular Life Sciences.

[33] Shiseki M, Nagashima M, Pedeux RM, Kitahama-Shiseki M, Miura K, Okamura S, Onogi H, Higashimoto Y, Appella E, Yokota J (2003). p29ING4 and p28ING5 bind to p53 and p300, and enhance p53 activity. Cancer research.

[34] Kim J-W, Jang S-M, Kim C-H, An J-H, Kang E-J, Choi K-H (2012). New molecular bridge between RelA/p65 and NF-κB target genes via histone acetyltransferase TIP60 cofactor. Journal of Biological Chemistry.

[35] Dey A, Tergaonkar V, Lane DP (2008). Double-edged swords as cancer therapeutics: simultaneously targeting p53 and NF-κB pathways. Nature Reviews Drug Discovery.

[36] Wadgaonkar R, Phelps KM, Haque Z, Williams AJ, Silverman ES, Collins T (1999). CREB-binding protein is a nuclear integrator of nuclear factor-κB and p53 signaling. Journal of Biological Chemistry.

[37] Patel JH, Du Y, Ard PG, Phillips C, Carella B, Chen C-J, Rakowski C, Chatterjee C, Lieberman PM, Lane WS (2004). The c-MYC oncoprotein is a substrate of the acetyltransferases hGCN5/PCAF and TIP60. Molecular and cellular biology.

[38] Heyne K, Winter C, Gerten F, Schmidt C, Roemer K (2013). A novel mechanism of crosstalk between the p53 and NFκB pathways: MDM2 binds and inhibits p65RelA. Cell Cycle.

[39] Hoffman B, Liebermann D (2008). Apoptotic signaling by c-MYC. Oncogene.

[40] Ho JS, Ma W, Mao DY, Benchimol S (2005). p53-Dependent transcriptional repression of c-myc is required for G1 cell cycle arrest. Molecular and cellular biology.

[41] Sherr CJ (2006). Divorcing ARF and p53: an unsettled case. Nature Reviews Cancer.

[42] Champagne KS, Kutateladze TG (2009). Structural insight into histone recognition by the ING PHD fingers. Current drug targets.

[43] Ullah M, Pelletier N, Xiao L, Zhao SP, Wang K, Degerny C, Tahmasebi S, Cayrou C, Doyon Y, Goh S-L (2008). Molecular architecture of quartet MOZ/MORF histone acetyltransferase complexes. Molecular and cellular biology.

