# Supplementary material for: The miR-193a-3p-regulated ING5 gene activates the DNA damage response pathway and inhibits multi-chemoresistance in bladder cancer
Source: Oncotarget. 2015 Mar 12;6(12):10195–206. doi: 10.18632/oncotarget.3555 (PMC4496349; doi:10.18632/oncotarget.3555)
Supplement: Supplementary file 1 [file oncotarget-06-10195-s001.pdf]

The miR-193a-3p-regulated ING5 gene activates the DNA damage response pathway and inhibits multi-chemoresistance in bladder cancer

Supplementary Material

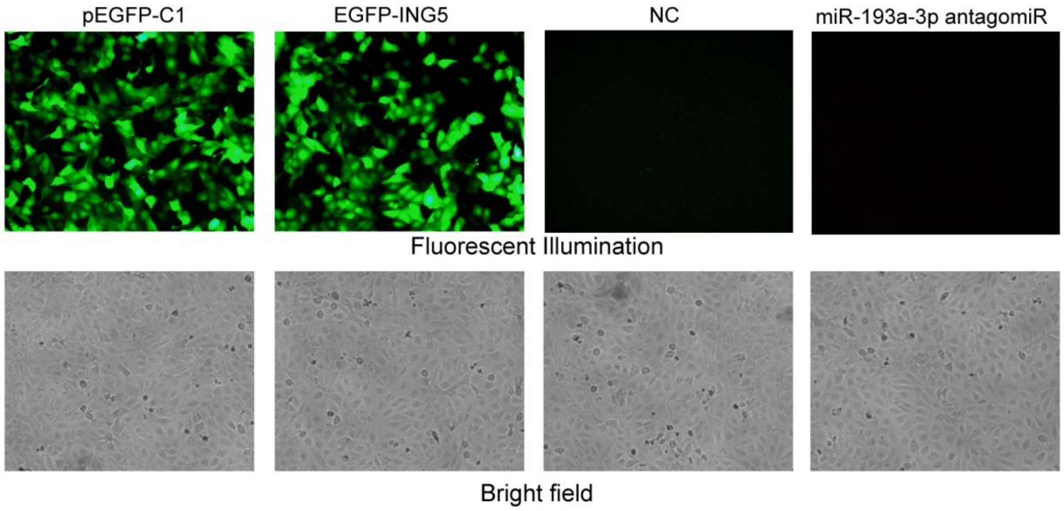

Supplementary Figure 1: The ING5 level in miR-193a-3p antagomiR- and overexpression construct-transfected H-bc cells. A representative field of H-bc cells transfected with miR-193a-3p antagomiR and the GFP-tagged ING5 ectopic expression construct is shown. Top panel: the green fluorescent illuminated field. Bottom panel: the bright field. The pEGFP-C1 vector and a nonsense microRNA sequence were used as negative controls.

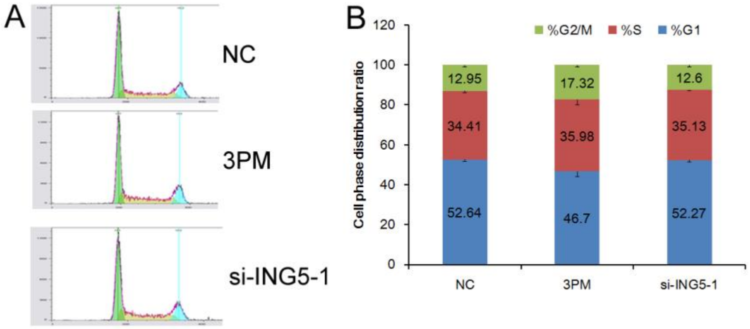

C

	%G1	%S	%G2/M
NC	52.64±0.77	34.41±0.98	12.95±1.1
3PM	46.7±2.8	35.98±2.6	17.32±0.99
si-ING5	52.27±0.98	35.13±0.22	12.6±1.2

Supplementary Figure 2: The effects of the forced reversal of both the miR-193a-3p and ING5 levels on the cell cycle profile of 5637 cells by FACS analysis. The percentages of cells in the G1 (blue), S (brown), and G2/M phases (green) compared with the original (**A**) is also shown in plot (**B**) and table (**C**) formats.

A

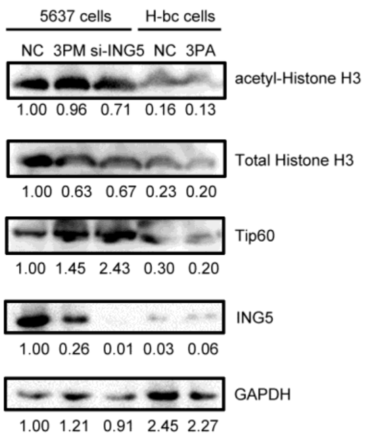

B

	5637 cells			H-bc cells	
	NC	3PM	Si-ING5	NC	3PA
Acetyl-H3/ total H3	1.00	1.52	1.06	0.70	0.65

	5637 cells			H-bc cells	
	NC	3PM	Si-ING5	NC	3PA
Tip60/ GAPDH	1.00	1.20	2.67	0.12	0.09

Supplementary Figure 3: ING5's effects on histone H3 acetylation and Tip60 level. **A**, Levels of acetyl-histone H3, total histone H3, Tip60 and ING5 in miR-193a-3p mimic- and ING5 siRNA-transfected 5637 cells and miR-193a-3p antagomiR-transfected H-bc cells compared with NC-transfected cells as assessed by Western blot. **B**, The ratio of the acetyl-histone H3 level to the total histone H3 level and the relative Tip60 protein level are calculated and summarized in the table.
